# Peculiarities of Uterine Cavity Biocenosis in Patients with Different Types of Endometrial Hyperproliferative Pathology

**DOI:** 10.25122/jml-2019-0074

**Published:** 2019

**Authors:** Natalia Ye Horban, Iraida B Vovk, Tamara O Lysiana, Inna H Ponomariova, Igor V Zhulkevych

**Affiliations:** 1. Institute of Pediatrics, Obstetrics and Gynecology of NAMS of Ukraine, Kiev, Ukraine; 2. I.Ya. Horbachevsky Ternopil National Medical University, Ternopil, Ukraine

**Keywords:** endometrial hyperplasia, endometrial polyp, microbiocenosis

## Abstract

The peculiarities of the microbiocenosis of the uterine cavity in 184 patients of reproductive age with different types of endometrial hyperproliferative processes were studied: Group 1, n=60, non-atypical endometrial hyperplasia (NAEH); Group 2, n=62, endometrial polyps (EPs); Group 3, n=62, NAEH + EPs. Correlation analysis of the degree of association between different biological media (uterus and genital tract) was carried out. Contamination of the uterine cavity by bacterial flora was diagnosed in all groups of patients examined. Anaerobic flora was about 30% while bacteria of the genus Bacteroides were the most common. Among aerobic organisms, representatives of the Enterobacteriaceae family and coccal flora are noted. The widest was the spectrum of isolated microorganisms in patients of group 2 (with endometrial polyps). A strong positive correlation was established between indices of genital tract contamination and the uterine cavity by coccal flora, Escherichia coli, and anaerobic organisms. The findings suggest dysbiotic changes and the presence of a chronic inflammatory focus in the endometrium, which pathogenetically substantiates the application of anti-inflammatory therapy in such patients.

## Introduction

Until recently, colonization of the upper genital tract by microbes was thought to be caused by the movement of microorganisms from the vagina through the cervix. The uterine cavity was regarded as sterile because cervical mucus, which has a high concentration of proinflammatory cytokines, immunoglobulins and antimicrobial peptides, provides a barrier effect [9]. Detection of microorganisms in the uterine cavity was associated with contamination of vaginal microflora samples, but high levels of colonization by endometrial microorganisms were detected in hysterectomy specimens in nonpregnant women [10, 12].

Current scientific evidence shows that there is an upward transport in the reproductive tract. In Zervomanolakis’ et al. study, radiolabelled human serum albumin macroaggregates corresponding to the size of a human sperm cell were prepared and made up a volume of 1-2 ml that was applied to the posterior vaginal fornix. After 2 minutes, the labeled particles were found in the uterus [17].

The study of Mitchell et al. involved 58 women who underwent hysterectomy [14]. Twelve bacterial species were studied by means of quantitative polymerase-chain-reaction (PCR). Vaginal samples were taken before hysterectomy, and samples from the uterine cavity were taken after hysterectomy. Colonization of the upper genital tract by at least one bacterial species was confirmed in 95% of cases. Lactobacillus spp. and Prevotella spp. were detected most commonly. It should be noted that the average bacterial count in the upper genital tract was 2-4 log10 rRNA-copies of genes per smear, lower compared to the vagina. These data indicate that the cervix acts as a partial filter for upward distribution of microorganisms, and the immune system reduces the bacterial load that can spread upward through the cervix.

According to some scientists, the presence of a small number of bacteria in the uterine cavity cannot be considered a pathology [10, 14]. Pathological processes in the endometrium can occur if there is a high concentration of bacteria in the uterus, in the case of mixed bacterial microflora and the presence of virulent strains.

The direct correlation between the clinical manifestations of acute inflammatory diseases of the uterine appendages with changes of natural resistance components and the presence of microorganisms in the fallopian tubes has been established, as well as the inverse correlation with the normal state of vaginal microbiocenosis [1].

It is also interesting that endometrial pathology was detected in 93.6% of patients with recurrent bacterial vaginosis in histological examination of endometrial tissue and hysteroscopic examination of the uterine mucosa [5]. The pathology spectrum included endometrial hyperplasia (58% of cases), pronounced chronic endometritis (47% of cases), endometrial polyps (42% of cases), chronic endometritis with endometrial hypoplasia (24% of cases).

An important discovery was the absence of sterility in the uterine cavity in both primary and recurrent bacterial vaginosis [1]. Determination in the composition of the microflora of the uterine cavity of representatives such as *Propionibacterium spp., Eubacterium spp., Peptostreptococcus spp., Bacteroides spp., Prevotella spp., Porphyromonas spp., Fusobacterium spp., Veillonella spp., Corynebacterium spp., Staphylococcus spp., Streptococcus spp., Enterococcus spp., Enterobacter spp., E. coli, Klebsiella spp., and Gardnerella vaginalis* associated with bacterial vaginosis is indicative of their participation in the development of inflammatory changes in the endometrium [7].

In patients with acute inflammation and during exacerbation of chronic inflammatory disease of the uterine appendages in the biotopes of the reproductive tract, an increase in the proportion or appearance of coagulase-positive staphylococci, enterobacteria, bacteroids, fusobacteria, peptostreptococci, propionic bacteria was noted [4, 6, 8]. The diversity of microbial taxa decreases in the opposite direction of the vagina (vagina>cervical canal>fallopian tubes). No microorganisms were isolated from the fallopian tubes in healthy women in the comparison group [3].

In the case of inflammatory diseases of the organs of the true pelvis, anaerobic gram-negative bacilli and mixed bacterial communities are often found in the uterine cavity and fallopian tubes. Hysteroscopy reveals positive results for the presence of microorganisms in the uterine cavity in almost 75% of patients diagnosed with chronic endometritis, and these microorganisms differ from the microorganisms found in the vagina [13, 15, 18].

Recent research by E. S. Pelzer et al. (2018) characterized the microbial community in the endometrium and endocervix in women with menorrhagia and dysmenorrhea [11]. Samples of paired endocervical and endometrial biopsies were selected from patients undergoing hysteroscopy and/or laparoscopy. The material was examined for microbial DNA. The data obtained indicated the presence of a small number of microbial populations different from each other in the endocervix and endometrium. Lactobacillus spp., present in all endocervical specimens, were most commonly detected. Microorganisms of the genera Prevotella, Fusobacterium and Jonquetella were also found. Scientists state that the upper portions of the female genital tract are not sterile. Differences were observed in the profiles of the endocervical microbial community compared with the endometrium, as well as in women with menorrhagia compared to patients with dysmenorrhea [11].

## Objective

Study of the frequency of bacterial agent manifestations in cultures from the uterine cavity of patients with non-atypical endometrial hyperproliferative pathology (NAEHP) and correlation analysis of the degree of association between different biological environments (uterine cavity and genital tract) in such women.

## Materials and Methods

The bacteriological examination involved 184 patients of reproductive age with NAEHP. Group 1 consisted of women with non-atypical endometrial hyperplasia (NAEH; n=60); group 2 – endometrial polyps (EPs; n=62); group 3 – patients with concomitant NAEHP (CNAEHP) namely UBP+NAEH; (n=62). Samples from the uterine cavity were obtained by scraping the walls of the uterine cavity during hysteroscopy, followed by transfer to a nutrient medium. Aerobic (staphylococcus, streptococcus, Escherichia coli, enterobacteria, Candida fungi and so forth) and anaerobic flora (lactobacilli, bacteroids) of uterine origin was investigated using a set of selective, differential nutrient media (Endo agar, egg-yolk salt agar (EYSA), blood agar, Sabouraud agar, Mueller-Hinton agar, MRS agar, thioglycollate medium). The number of all bacterial strains in 1 ml of sample was determined by the number of colonies grown, taking into account the degree of dilution of the cultured material in ratio to the standard and transferring the data to decimal logarithms. 104 CFU/ml was considered to be the diagnostic concentration in the genital tract. Regarding the content from the uterine cavity, any results of the growth of bacterial agents in nutrient media (from 102 CFU) were evaluated. Anaerobic microflora was studied in “Anaerocult” anaerostat (Merck, Germany) and identified using Mikro La Test “Anaerotest 23” kits (Erba Lachema, Czech Republic). Bacterial vaginosis was diagnosed with the help of bacterioscopy by means of Romanowsky staining technique, followed by the counting of “key” cells, performing the amine and pH test. MIKPOLA-TEST “Candida test 22” kit (Erba Lachema s.r.o.; Czech Republic) was used to identify isolated fungal species. Any results of the growth of bacterial agents on nutrient media (from 102 CFU) were evaluated.

### Data processing

Statistical processing was performed using MedStat. The chi-squared test was used to compare the frequency of manifestation of the trait [16]; in all cases, the critical level of significance was assumed to be 0.05. Spearman’s rank correlation coefficient was also determined.

## Results and Discussion

The results of bacteriological studies of the samples from the uterine cavity in women of reproductive age with non-atypical hyperproliferative pathology of the endometrium showed registration of different species of microflora in all examined patients (Table 1).

The spectrum of aerobic gram-positive microflora isolated from the uterine cavity of patients with non-atypical endometrial hyperplasia most often included Str. faecalis – in 20.0% (12/60) of patients, Str. аgalactiae – in 13.3% (8/60), St. epidermidis with hemolytic properties – in 16.7% (10/60) of women and St. epidermidis – in 11.7% (7/60) of cases. Among enterobacteria, the highest incidence of contamination of the uterine cavity by E. coli was reported in 21.7% (13/60) of patients. Enterobacter spp. were cultured with less frequency in 11.7% (7/60) of women and Klebsiella spp. – in 6.7% (4/60) of patients. Candida fungi were isolated from the uterine cavity in only 5.0% (3/60) of the examined patients. Monocultures of different types of microflora were identified in 23 (38.3%) patients, two-component associations – in 17 (28.3%) women, and 5 (8.3%) patients had three-component associations. It should be noted that the quantitative indices of culturing aerobic opportunistic microflora did not reach a high level (102-104 CFU/ml).

Anaerobic microflora isolated from the uterine cavity in women with NAEH was most commonly represented by Bacteroides spp. - in 30.0% (18/60) of patients and Peptostreptococcus spp. - in 18.3% (11/60) of patients. The spectrum also included Prevotella spp. – in 8.3% (5/60) of the examined patients, Fusobacterium spp. – 6.7% (10/60), Peptococcus spp. – 15% (9/60), Veillonella spp. – 13.3% (8/60) and Eubacterium spp. – 10.0% (6/60). Quantitative indices of culturing obligate anaerobes from the uterine cavity in patients of this group did not reach a high level either (102-104 CFU/ml).

In the second group of women (with endometrial polyps), a higher level of bacterial contamination was diagnosed in the samples obtained from the uterine cavity during surgical treatment compared to the patients in the first group (with NAEH).

Aerobe organisms (isolated from endometrial tissue of women in group 2) were predominantly represented by cocci of different taxa: Str. faecalis – in 21.0% (13/62) of patients, Str. galactiae – 19.4% (12/62), St. aureus – 17.7% (11/62), St. epidermidis – 25.8% (16/62), St. epidermidis with hemolytic properties – 22.6% (14/62) and enterobacteria (E. coli – 22.6% (14/62), Enterobacter spp. – 12.9% (8/62) and Klebsiella spp. – 8.1% (5/62). The frequency of fungi of the genus Candida was 6.5% (4/62). The concentration of isolated aerobic potentially pathogenic microflora in the samples from the uterine cavity was slightly higher than the indices obtained in patients in the group with NAEH but statistically had no reliable difference.

Regarding anaerobic microflora, Bacteroides spp., the predominant species, isolated from the uterine cavity in patients in group 2 (with EPs), as well as in women of group 1 (with NGE), were found in 38.7% (24/62) of cases. There has also been some increase in the content of other species of this flora: Peptostreptococcus spp. – in 24.2% (15/62) of patients, Prevotella spp. –19.4% (12/62), Fusobacterium spp. – 25.8% (16/62) and Eubacterium spp. – 22.6% (14/62).

Quantitative indices of culturing anaerobic microflora from the uterine cavity, with the exception of bacteroids, in women with EPs were at a higher level (mostly 104 CFU).

Bacteriological examination of the samples from the uterine cavity in women of group 3 (with CNAEHP) revealed a slight increase in the frequency of manifestation of aerobic opportunistic microorganisms: St. epidermidis and Str. agalactiae were identified in 22.6% (14/62) of patients, St. aureus in 21.0% (13/62) and E. coli in 30.6% (19/62). Quantitative indices of culturing aerobic microorganisms constituted 102-104 CFU/ml. The frequency of culturing fungi of the genus Candida was 11.3% (7/62), being detected in the amount of 104-106 CFU/ml, which was a higher rate compared to patients in other groups.

The results of the analysis of anaerobic microflora from the uterine cavity in patients of group 3 indicate some decrease in individual indices of their culturing compared with the data obtained in women of group 2 (with EPs): Bacteroides spp. were detected in 33.9% (21/62) of patients, but in a smaller number (102 CFU/ml) than in patients in other groups. The frequency of culturing other representatives of anaerobic microorganisms from the uterine cavity in patients of group 3 (with CNAEHP) was: Peptostreptococcus spp. – in 17.7% (11/62) of patients, Prevotella spp. – 16.1% (10/62), Fusobacterium spp. – 24.5% (15/62) and Veillonella spp. – 21.0% (13/62) . The anaerobic concentration was 102-104 CFU/ml, as in the vast majority of patients in other groups.

Consequently, the results of the bacteriological studies of the samples from the uterine cavity in women of group 3 (with CNAEHP) indicate significant changes in the quantitative and qualitative parameters of microbiocenosis. Although there is currently little information on the association between changes in the uterine flora and endometrial hyperproliferative pathology, previous scientific studies have shown that bacteria may play a role in the development of hyperplasia by stimulating proliferation or by inhibiting cell apoptosis [2, 15].

Summarizing the data, we can say that in the group of women with NAEHP, anaerobic microorganisms Bacteroides spp. were most common, namely in 30.0% (18/60) of patients. Aerobic flora was represented by E. coli – 21.7% (13/60) and Str. faecalis – 20.0% (12/60).

In patients of group 2 (with EPs) the spectrum of isolated microorganisms from the uterine cavity was wider, but also predominantly represented by anaerobic bacteria – Bacteroides spp. – 38.7% (24/62), Fusobacterium spp. – 25.8% (16/62), Peptostreptococcus spp. – 24.2% (15/62), Veillonella spp. and Eubacterium spp. – 22.6% (14/62). Among the aerobic organisms, there was a high frequency in St. epidermidis – 25.8% (16/62), St. epidermidis with hemolytic properties and E. coli - 22.6% (14/62) and Str. faecalis – 21.0% (13/62).

In women of group 3, the culture was represented by Bacteroides spp. – 33.9% (21/62), E. coli – 30.6% (19/62) and Str. faecalis – 24.2% (15/62), results which were similar to those obtained in women of group 1 (with NAEH). However, the incidence of some other species was quite high: Fusobacterium spp. – 24.2% (15/62), St. epidermidis and Str. agalactiae – 22.6% (14/62), St. aureus, Veillonella spp. and Eubacterium spp. – 21.0% (13/62), which was characteristic for women of group 2 (with EPs).

Using correlation analysis of the degree of association between the microflora of different biological media in women with NAEHP, we found that in these patients of reproductive age, a strong positive correlation between the indices of contamination of the genital tract and the uterine cavity by coccal flora (r=0.801, r=0.754, r=0.928, r=0.904), Escherichia coli (r=0.905) and anaerobic flora (r=0.806, r=0.736, r=0.715) were also observed (Table 2).

In women of group 2 (with EPs) there was a strong positive correlation between the indices of genital contamination and the uterine cavity by coccal flora (r=1, r=0.859), Escherichia coli (r=0.736), Enterobacter (r=0.819) and anaerobes (r=0.924, r=0.763, r=0.836, r=0.992, r=0.994) (Table 3).

In contrast to the patients of group 1, in patients of group 2, there was no strong positive correlation between the contamination of reproductive tract and the uterine cavity by Streptococci (r=0.744 and r=0.601), Enterobacter (r=0.587) and anaerobes (r=0.678). It should also be noted that during the study, no association was found between the genital tract and uterine cavity contamination by Candida fungi and the presence of NAEH or Eps.

It should be noted that, most frequently, a strong positive correlation between the indices of the genital tract and the uterine cavity contamination was found in patients of group 3 with concomitant non-atypical endometrial hyperproliferative pathology (Table 4).

The presence of a moderate positive correlation in the contamination of the genital tract and uterine cavity by fungi of the genus Candida (r=0.519), which was not detected in the former groups of the examined patients, was an interesting discovery.

Therefore, in our study, when the cultures from the uterine cavity in women with NAEHP were studied, anaerobic flora accounted for about 30.0% of all isolated microorganisms, among which gram-negative bacteria of the genus Bacteroides prevailed. Representatives of the Enterobacteriaceae family, in particular, E. coli and gram-positive cocci (Staphylococci and Streptococci) prevailed among the aerobic microorganisms.

The presence of bacteria from the Enterococcaceae family and other opportunistic microorganisms in their vaginal biotope and uterine cavity may indicate a translocation process from the intestinal biotope against the background of mucosal colonization resistance in women with NAEHP.

The wide range of isolated representatives of anaerobic microflora of the Enterobacteriaceae family in the examined patients is indicative of dysbiotic changes in the studied biotopes, and the detection of Staphylococcus aureus, which is a pathogen, indicates the presence of chronic inflammation foci (chronic course of the disease) in the woman’s body.

## Conclusions

In all groups of patients with non-atypical hyperproliferative pathology of the endometrium, contamination of the uterine cavity with bacterial flora is registered.

Anaerobic flora accounted for about 30.0% of all isolated microorganisms, among which gram-negative bacteria of the genus Bacteroides spp. prevailed. The representatives of the Enterobacteriaceae family, in particular, E. coli and gram-positive cocci (staphylococci and streptococci) prevailed among aerobic microorganisms.

The spectrum of isolated microorganisms from the uterine cavity was the widest in patients with endometrial polyps.

It is established that patients with NAEHP demonstrate a strong positive correlation between the indices of microbial contamination of the genital tract and the uterine cavity with coccal flora, Escherichia coli, anaerobic organisms, indicating the presence of chronic inflammation foci in the endometrium, and requires complex anti-inflammatory treatment in such a contingent of women.

## Conflict of Interest

The authors confirm that there are no conflicts of interest.
